# Application of Knowledge Distillation Based on Transfer Learning of ERNIE Model in Intelligent Dialogue Intention Recognition

**DOI:** 10.3390/s22031270

**Published:** 2022-02-08

**Authors:** Shiguang Guo, Qing Wang

**Affiliations:** School of Information & Communication Engineering, Beijing Information Science and Technology University, Beijing 100025, China; qingwang@bistu.edu.cn

**Keywords:** ERNIE, deep learning, distill, chatbot

## Abstract

The ‘intention’ classification of a user question is an important element of a task-engine driven chatbot. The essence of a user question’s intention understanding is the text classification. The transfer learning, such as BERT (Bidirectional Encoder Representations from Transformers) and ERNIE (Enhanced Representation through Knowledge Integration), has put the text classification task into a new level, but the BERT and ERNIE model are difficult to support high QPS (queries per second) intelligent dialogue systems due to computational performance issues. In reality, the simple classification model usually shows a high computational performance, but they are limited by low accuracy. In this paper, we use knowledge of the ERNIE model to distill the FastText model; the ERNIE model works as a teacher model to predict the massive online unlabeled data for data enhancement, and then guides the training of the student model of FastText with better computational efficiency. The FastText model is distilled by the ERNIE model in chatbot intention classification. This not only guarantees the superiority of its original computational performance, but also the intention classification accuracy has been significantly improved.

## 1. Introduction

Chatbots have become more widely used in industries and smart cities with the rapid development of Artificial Intelligence. Chatbots are generally divided into either open-domain and closed-domain. In an open context, users can chat with chatbots on any topic such as entertainment, emotional companionship, and socialization, which is known as Chitchat bots. In a closed context, you can only talk about the topic set by the robot, which belongs to the closed-domain type of chatbots. In addition, closed-domain chatbots are designed to accomplish a certain task, such as customer support and shopping guidance, which is known as a task-oriented chatbot. For example, Ali Xiaomi [1] is an Intelligent Personal Assistant in the field of e-commerce; based on Ali’s massive consumption and business data, combined with the needs of online and offline life scenarios, it provides intelligent shopping guides, services, and assistants with an anthropomorphic interactive business experience in a smart manual mode. An open-domain chatbot, such as Siri from Apple or Xiaobing [2] from Microsoft, can provide a 24 h personal assistant for users.

**Figure 1:**
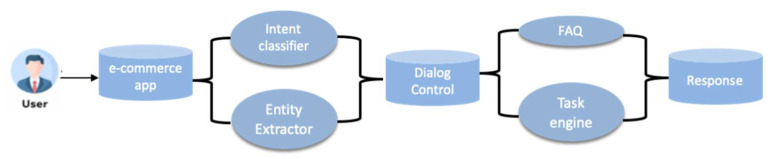
Workflow of an e-commerce chatbot.

Intention classification is essential to the performance of a task-oriented chatbot. The user’s intention determines the success of the subsequent chatbot process, as shown in Figure 1. The core of intention recognition is text classification, which can be regarded as a traditional problem in the field of natural language processing. Traditional machine learning methods for text classification include the support vector machine (SVM) [3]; however, it was limited by low accuracy for the text classification task. In recent years, with the rapid development of deep learning technology, neural networks have been applied to text classification technology. Convolution neural network (CNN) has achieved great success in the field of images [4]. ColloBERT applied CNN to natural language tasks. The TextCNN [5] applied CNN to text classification with effective results. Since CNN cannot capture long-distance dependence, and the Recurrent Neural Network (RNN) [6] can handle time series features, RNN has shown advantages in natural language processing and feature extraction. To solve the gradient disappearance problem of RNN in the training process, an RNN-based variant of the Long-Short Term Memory (LSTM) network and GRU (Gate Recurrent Unit) network structure [7] have been proposed. Additionally, there is an advance for multivariate time series features extraction [8].

RNN-based models are limited by performance issues, and it is difficult to have beneficial real-time applications, such as high-concurrency scenarios in the field of chatbots. Yang et al. [9] discussed the application of deep learning in the intention classification of chatbots. Bahdanau et al. in 2015 [10] developed an attention mechanism, while Vaswani et al. in 2017 [11] proposed a transformer as seq2seq model with parallelized attentions. In 2017, Mikolov et al. [12] proposed the FastText model; the network architecture of FastText is very similar to CBOW (Continuous Bag-of-Words) [13], and FastText is mainly used for text classification and displays positive computational efficiency. Peters et al. [14] generalizes traditional word embedding research ELMo (Embedding from Language Model) along a different dimension in 2018, and they suggested extracting context sensitive features and applying pre-training tasks to text classification tasks.

Fine-tuning based on pre-training for a language model has been developed for text generation tasks (e.g., GPT (Radford et al., 2018) [15]; Google proposed the BERT (Bidirectional Encoder Representations from Transformers) model in 2018 [16]. The BERT model is based on a self-attention model architecture. The successful application of BERT has put the natural language text classification task into a new level.

The BERT model mainly focuses on words or more fine-grained cloze learning and does not make full use of the lexical structure in the training data. Li [17] used the application of the BERT model in text classification at the sub-word level. Baidu proposed the ERNIE (Enhanced Representation through Knowledge Integration) model in 2018 [18]. Compared with BERT, ERNIE has improved two masking strategies; the first is the phrase-based masking strategy, and the second is the entity (name, location, organization, product, etc.) based masking strategy. Although the BERT and ERNIE models can achieve positive results in various tasks in the field of natural language processing, it is difficult for scientific research teams in universities as well as in small- and medium-sized enterprises to pre-train BERT from the base model due to the large size of the model, the large amount of data, and the large number of resources required for training. In the field of computer vision, Hinton et al. [19] proposed a training method based on the “teacher-student network idea” in 2015. The basic principle was to transfer the features learned from the “teacher” network with a complex network structure and strong learning ability to the “student” network with a small number of parameters and weak learning ability. Sau et al. [20] used a noise-based regularizer to train the student from the teacher for a higher training efficiency in 2016. Liao et al. [21] tried to use the BERT model for distillation learning in LSTM and CNN. Sun et al. [22] proposed Patient Knowledge Distillation for the BERT Model.

In e-commerce, it is usually computationally expensive when the chatbot handles a large number of real customers online; the simple intention classification model can show a beneficial computational performance but is limited to the low classification accuracy. To improve the performance of the small-scale model, the knowledge obtained by the “teacher” model can guide the training of the original small-scale model to achieve better results. In this study, we develop a chatbot for after-sale service in e-commerce to help manage the high computational price issue. The pre-trained ERNIE model will distill the FastText model to increase the efficiency and maintain the classification accuracy. Through the knowledge distillation method, the teacher model ERNIE is used to predict intention labels on a large amount of online data, and the student model FastText can be distilled through the enhanced data of large-scale corpus from the teacher model. With these large amounts of unlabeled text, the knowledge in the teacher model ERNIE can be better transferred to the student model FastText. In addition, several different text classification models, such as LSTM and BERT, are performed to the same task for comparison.

## 2. Pre-Trained Model

Pre-training models have long been used in the field of computer vision. The transfer learning is pre-training a neural network model on a known task, and then performing fine-tuning using the trained neural network as the basis to a new purpose-specific model. Recently, in the field of natural language processing (NLP), the use of language model pre-training methods has achieved beneficial improvements on multiple NLP tasks. These tasks include natural text classification, sentence comprehension, and other sentence-level tasks.

There are two strategies for applying pre-trained language features to tasks: feature-based and fine-tuning. For example, ELMo uses pre-trained features as additional features; a pre-trained neural network produces word embeddings, which are subsequently used as a feature on NLP models. Fine-tuning is to apply the learning ideas of transfer learning to the deep learning language model. The network model and parameter weights are determined mainly through pre-training. In practical applications, the pre-trained model is selectively loaded and then a new data set is added to retrain the model; this is called the basic step of fine-tuning, such as BERT.

**Figure 2:**
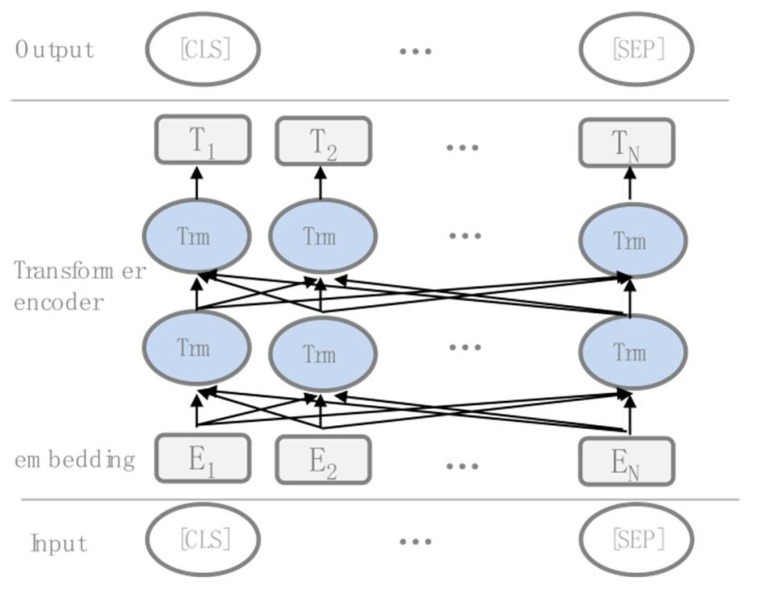
Structure of BERT model.

BERT makes use of Transformer, an attention mechanism that learns contextual relations between words (or sub-words) in a text. The BERT model proposed a new pre-training target: the masked language model (MLM). The models structure is shown in Figure 2. MLM randomly selects various words to mask, with the purpose to predict the masked portion based on the context non-masked words in the sequence, the MLM can access future context information due to the bidirectional feature provided by the Transformer architecture. In addition, BERT performs the next sentence prediction task, and the model receives pairs of sentences as input and learns to predict if the second sentence in the pair is the subsequent sentence in the original corpus.

## 3. Knowledge Distillation

**Figure 3:**
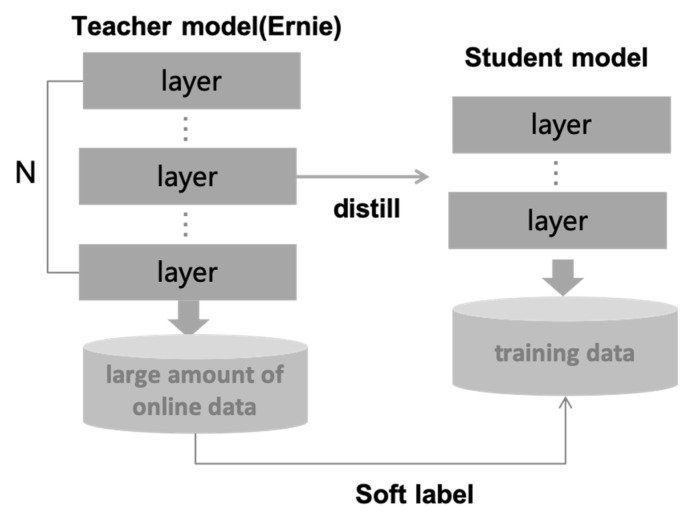
Intention classification framework based on knowledge distillation.

The knowledge distillation is teaching a smaller model that is more suitable for reasoning by using an already trained network; the process by which is also known as model compression. Knowledge distillation introduces the soft target of the “teacher” model with more complex reasoning ability to teach the “student” network with small parameters and weak learning ability, as shown in Figure 3. The soft label is the probability vector output for large-scale samples, and the probability vector has more information compared with the one-hot label for text classification task. By passing the soft label information of the large model to the student model, the learning ability of the student model can be improved.

Due to the large-scale softmax output, the value distributed in the correct position will be large, while the value of other positions will be small. It is often more effective to directly use the probability distribution of the teacher network to learn the student network. It is necessary to improve the softmax function, which is called a heating technique.

Original softmax function:

(1) $$y_{i}=\frac{\exp(x_{i})}{\sum_{j=1}^{N}\exp(x_{j})}$$

where, *y_i_* is the predicted probability that the document belongs to in the *j*-th class, *x_i_* denotes the elements of the input vector to the softmax function, and *N* represents the number of classes in the multi-class classifier. The term on the bottom of the formula is the normalization term.

Improved softmax function:

(2) yi=exp(xiT)∑j=1Nexp(xjT)

where *T* is the temperature with a fixed value.

## 4. Teacher Model (ERNIE)

**Figure 4:**
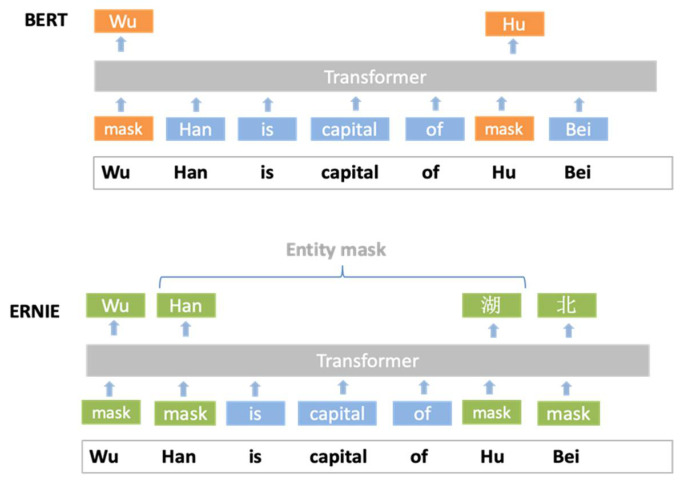
BERT and ERNIE mask mechanism.

Compared with BERT, ERNIE 1.0 has improved two masking strategies, the first based on the phrase, and the second based on the entity (name, location, organization, product, etc.), such as Beijing. In ERNIE, the phrase or entity composed of multiple words is regarded as a unified unit. Compared with the word-based mask of BERT, ERNIE is trained through the mask of the entity unit. In contrast to directly mapping knowledge queries into vectors and then adding them up directly, ERNIE can potentially learn knowledge dependence and longer semantic dependence through a unified mask to make the model more generalized, as shown in Figure 4.

In addition, ERNIE 2.0 builds lexical level, grammatical level, and semantic level pre-training tasks based on ERNIE1.0 and accumulates new knowledge through multi-tasking and continuous learning. In this task, ERNIE1.0 base is used as the teacher model.

## 5. Student Model (FastText)

**Figure 5:**
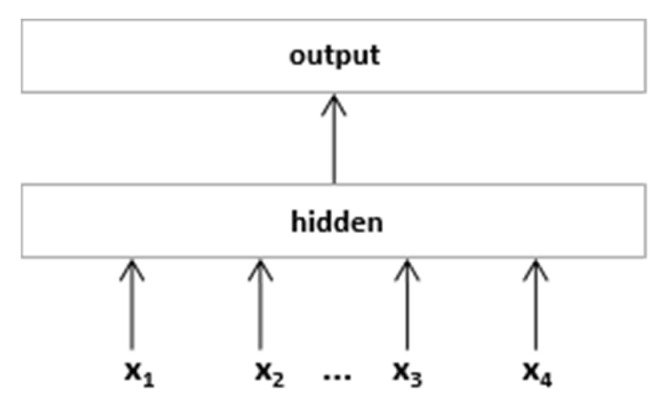
FastText model mechanism.

FastText’s network architecture is very similar to CBOW; it is mainly used for text classification compared to CBOW. For text classification of short text, short text sentences are represented by a “bag of words” model, and then linear classifiers are used for text classification. FastText shows a positive computational performance in text classification tasks based on its network structure. The input layer of the FastText network model (Figure 5) is the word embedding vector of the word sequence of a sentence or a paragraph of text.

These feature vectors are averaged to generate text vectors. The output layer uses hierarchical softmax, which uses the frequency of the classification label to make the leaf nodes of HuffmanTree. Each node is associated with a probability, and the probability value is equal to the product of the probability from the root node to the leaf nodes, which belong to this class. If the depth of the leaf nodes is l+1, their parent node distribution is $n_{1},n_{2},\cdots ,n_{l}$.

Its probability can be expressed as:

(3) $$P(n_{l+1})=\prod_{i=1}^{l}P(n_{i})$$

where, *P*(*n_i_*) denotes the conditional probability of the next word in a sequence.

To optimize the calculation, the hierarchical softmax model is adopted. The Huffman Tree is constructed by using the occurrence frequency of the term or classification label, which is used as the leaf node of the Huffman Tree. The input of CBOW is the term in the context window, and the output layer corresponds to the term with the highest probability. The input of FastText is the content of the entire sentence, including term and *n* = gram. The output layer corresponds to the classified label. The purpose of CBOW is to obtain the required word vector through repeated iterations. FastText traverses all nodes of the tree and finds the classification label with the highest probability.

## 6. Experiment and Result Analysis

It mainly used the after-sales intelligent customer service Q&A of real e-commerce as the corpus, focusing on 16 task-oriented intention recognitions. The training corpus was manually labeled, with 120,000 corpora, as shown in Table 1. In the 120,000 corpora, there were 15 customer business intentions, which mainly included the return of goods, urging delivery, request of a replacement, application for an exchange, etc. The 16th intention was other categories (non-business intentions), which mainly included chitchat-related corpus. This corpus had an average of 7000 samples per business category. In addition, there were about 10,000 test samples as the test set.

**Figure 6:**
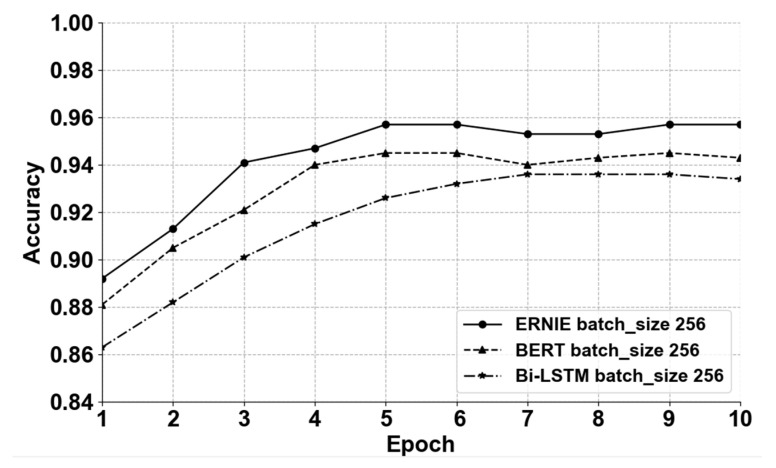
Accuracy of different algorithms.

In the experiment, the different models were used to learn the training corpus. The specific training parameters of BERT and ERNIE are shown in Table 2, with a total of 12 layers and a hidden layer of 768 dimensions. The 12-head model was used (Table 2), with a total of 110 million parameters. Next, the optimized different models were used to evaluate the test data separately, as shown in Figure 6. The ERNIE model archived best the performance of accuracy with increments of the epoch.

Next, we used the ERNIE base model as the teacher model, while the FastText model was used as the student model for training with different dimensions. The size of ngram was set to 2 (Table 3).

**Figure 7:**
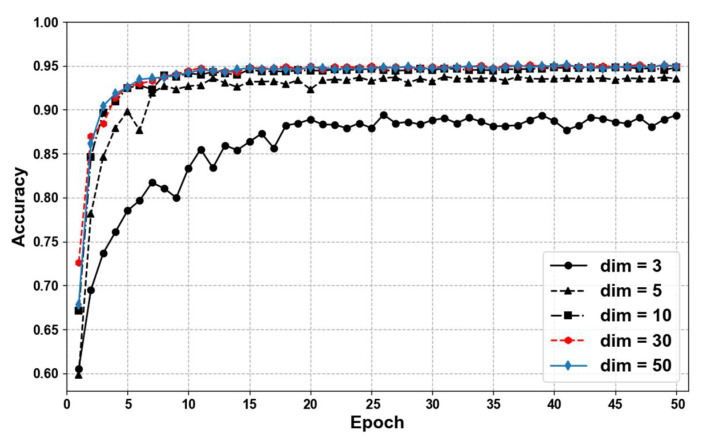
Accuracy of Epoch with different dimensions of FastText model (-ngrams = 2; learning rate = 0.01; neg = 10).

**Figure 8:**
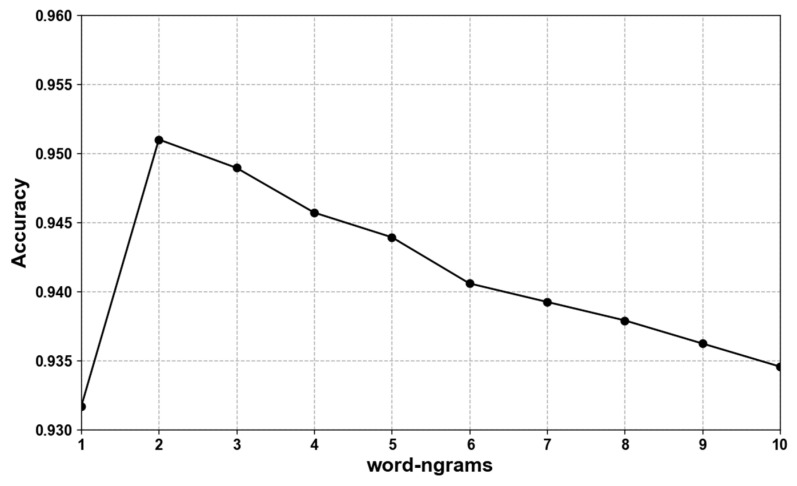
Accuracy at different -ngrams of FastText model (dimensions = 2; learning rate = 0.01; neg = 10).

One million online real customer service chat corpora were used for distillation. The teacher model was used to obtain the tags, and next the student model FastText was distilled. We conducted several FastText experiments with different dimensions, as shown in Figure 7, where the accuracy increased with larger dimension numbers. The best performance of accuracy reached 0.95, which did not surpass the teacher model ERNIE in its accuracy of 0.957. In addition, the experiments of different word-ngrams was also conducted, as shown in Figure 8. This experiment showed which word-ngrams reached the best accuracy performance of 0.951, of which there were two.

In addition, the LSTM and BERT algorithm models were used for intention classification on the same corpus for comparison with the same training and test corpus. The results of Bi-LSTM, BERT base, ERNIE base, FastText, and FastText (ERNIE distillation) are shown in Table 4.

It can be seen from the experimental results that the FastText model has the lowest accuracy before distillation, but it shows the best computational performance. For the Chinese intention classification problem of e-commerce customer service, the ERNIE (base) model has a significant improvement in accuracy when compared with the BERT (base) model. There is not much difference in the average time consumption of online services between these two models. FastText distilled by ERNIE (base) has a significant improvement in accuracy, 95%, with a beneficial computational performance.

The model complexity and the number of parameters of FastText have no advantages over the ERNIE model, but it has obvious advantages in computational performance and has a beneficial performance of accuracy after distillation.

## 7. Conclusions

This paper applied the teacher-student distillation model, as well as proposed the teacher model based on ERNIE. Further, it applied it to the student model based on FastText in its intention recognition task of a chatbot for after-sale services in e-commerce. Compared with traditional models, the ERNIE model based on transfer learning had a beneficial advantage in the task of intelligent dialogue intention classifications. However, the network structure of the ERNIE model was complex, and it was difficult to meet the online performance requirements under the condition of high concurrent qps in large-scale e-commerce scenarios. In addition, GPU was expensive and had no cost advantage. Therefore, the labeling inference of the ERNIE model was performed on one million online corpora, and then the distillation was carried out on the student model FastText by using the one million online labeled corpora labeled by teach model of ERNIE.

Experiments on real chatbot corpus demonstrated that the ERNIE model outperformed the other models, such as LSTM, FastText, and BERT. The intention recognition accuracy of the ERNIE model could reach 95.7% in 16 tasks of 100,000 intelligent dialogue tasks, which was higher than LSTM and BERT in the same task. By using ERNIE as the teacher model and distilling the student model based on FastText, the intention recognition accuracy was improved by about 0.94%, compared with the native FastText model. The performance of FastText could reach 0.1 ms, which could meet the online requirements of high concurrency.

## Figures and Tables

**Table 1:** Part of the training corpus.

Corpus	Classification
I haven’t received the goods for so long.	Urging Delivery
Why haven’t my shoes arrived for so long?	Urging Delivery
Haven’t my things arrived yet?	Urging Delivery
When will the exchanged goods come?	Check the Replacement Progress
When will you send me a replacement?	Check the Replacement Progress
When to exchange?	Check the Replacement Progress
Where do I apply for exchange?	Request a Replacement
I can’t find the place to apply for a replacement.	Request a Replacement
The two pairs of children’s shoes I bought yesterday are small, how could I change them?	Request A Replacement
Send it back by myself. What about the return shipping fee?	Return Shipping Fee
How to calculate the return shipping fee for self-return?	Return Shipping Fee
How to deal with the return shipping fee for self-return?	Return Shipping Fee

**Table 2:** Super parameters of ERNIE and BERT model training.

ERNIE and BERT Parameters	Value
Layer Numbers	12
Learning Rate	5 × 10^−5^
Batch Size	256
Weight Decay	0.01
Epoch	10

**Table 3:** Parameters of FastText model training.

FastText Parameters	Value
Learning Rate	0.01
neg	10
ngram	2
Dimension	30

**Table 4:** Comparison of accuracy and time-consuming of different models.

Model	Accuracy (%)	Time-Consuming (ms)
Bi-LSTM	94.36	10.0+
FastText	94.16	0.1
BERT (Base)	94.50	10.0+
ERNIE (Base)	95.70	10.0+
FastText (ERNIE Distillation)	95.10	0.1

## Data Availability

Not applicable.
